# Online Statistical Learning in Developmental Language Disorder

**DOI:** 10.3389/fnhum.2021.715818

**Published:** 2021-09-27

**Authors:** Ágnes Lukács, Krisztina Sára Lukics, Dorottya Dobó

**Affiliations:** ^1^Department of Cognitive Science, Budapest University of Technology and Economics, Budapest, Hungary; ^2^ELKH-BME Momentum Language Acquisition Research Group, Eötvös Loránd Research Network (ELKH), Budapest, Hungary

**Keywords:** statistical learning, developmental language disorder, acoustic segmentation, visual segmentation, online and offline measures

## Abstract

**Purpose**: The vulnerability of statistical learning (SL) in developmental language disorder (DLD) has mainly been demonstrated with metacognitive offline measures which give little insight into the more specific nature and timing of learning. Our aims in this study were to test SL in children with and without DLD with both online and offline measures and to compare the efficiency of SL in the visual and acoustic modalities in DLD.

**Method**: We explored SL in school-age children with and without DLD matched on age and sex (*n* = 36). SL was investigated with the use of acoustic verbal and visual nonverbal segmentation tasks relying on online (reaction times and accuracy) and offline (two-alternative forced choice, 2AFC and production) measures.

**Results**: In online measures, learning was evident in both groups in both the visual and acoustic modalities, while offline measures showed difficulties in DLD. The visual production task showed a significant learning effect in both groups, while the visual two-alternative forced choice (2AFC) and the two acoustic offline tasks only showed evidence of learning in the control group. The comparison of learning indices revealed an SL impairment in DLD, which is present in both modalities.

**Conclusions**: Our findings suggest that children with DLD are comparable to typically developing (TD) children in their ability to extract acoustic verbal and visual nonverbal patterns that are cued only by transitional probabilities in online tasks, but they show impairments on metacognitive measures of learning. The pattern of online and offline measures implies that online tests can be more sensitive and valid indices of SL than offline tasks, and the combined use of different measures provides a better picture of learning efficiency, especially in groups where metacognitive tasks are challenging.

## Introduction

The complex process through which infants become proficient language users relies on a number of cognitive functions. Such functions are, among others, auditory processing, verbal short-term memory capacity, attention, categorization and mentalization (see e.g., Tomasello, 2003). Several studies have shown that the ability to learn structured patterns and extract statistical regularities (e.g., the frequent co-occurrence of syllables in words, word form-meaning pairs, grammatical relations) also plays a crucial role in language acquisition. This capacity is usually referred to as statistical learning (SL; Saffran et al., 1996a; Frost et al., 2015; Siegelman et al., 2017; Conway, 2020).

The substantial role of SL in language acquisition has been demonstrated by studies showing associations of SL with lexical knowledge (Yu, 2008; Spencer et al., 2015), sentence comprehension (Kidd, 2012; Misyak and Christiansen, 2012; Kidd and Arciuli, 2016) and language related skills such as reading and writing (Nicolson and Fawcett, 2011; Arciuli and Simpson, 2012; Arciuli, 2018). In accordance with these findings, research in atypical populations have shown SL impairments in individuals with language or literacy problems such as developmental dyslexia (e.g., Pavlidou et al., 2010; Gabay et al., 2015; Kahta and Schiff, 2016; Sigurdardottir et al., 2017; for a meta-analysis see: van Witteloostuijn et al., 2017) or developmental language disorder (DLD; e.g., Evans et al., 2009; Hedenius et al., 2011; Hsu et al., 2014; Lukács and Kemény, 2014; Haebig et al., 2017; Lammertink et al., 2020a; for meta-analyses see: Obeid et al., 2016; Lammertink et al., 2017). Although linguistic impairment is frequently associated with SL deficits, it is not yet clear whether SL is causal in language problems.

### Statistical Learning in Developmental Language Disorder

Individuals with DLD exhibit difficulties in learning and using their mother tongue, which are not accounted for by neurological problems, sensory disorders, environmental causes, or low general intelligence or social abilities. The phenotypic heterogeneity among individuals with DLD is associated with etiological heterogeneity, in which both genetic and environmental risk factors play an important role, and the interaction of these factors leads to DLD. However, the etiological factors are not completely known yet. Difficulties are present in multiple areas of language: children with DLD perform below age expectations on phonological, morphological, lexical and syntactic tasks as well (for a review see e.g., Leonard, 2014). Research on linguistic abilities of children with DLD have emphasized the heterogeneity of linguistic skills in this disorder (for thinking of DLD as a spectrum disorder see: Lancaster and Camarata, 2019), and studies revealed high variability in nonlinguistic cognitive capacities as well, including for example working memory (e.g., Montgomery, 2003; Montgomery et al., 2010), motor skills (Hill, 2001) and executive functions (Henry et al., 2012; Lukács et al., 2016; Kapa et al., 2017).

As discussed above, the impairment of SL in DLD is amply documented in different areas. Impairments have been found in word segmentation (in the extraction of word boundaries by relying on difference between transitional probabilities between the adjacent syllables; e.g., Evans et al., 2009; Haebig et al., 2017), non-adjacent dependency learning (extracting patterns, when two or more, temporally or spatially separated elements are related to each other; e.g., Hsu et al., 2014; Lammertink et al., 2020a), artificial grammar learning (Lukács and Kemény, 2014), motor sequence learning (Hsu and Bishop, 2014; Mayor-Dubois et al., 2014; for a meta-analysis see Lum et al., 2014) and probabilistic categorization (Kemény and Lukács, 2009; but see Lukács and Kemény, 2014). While the SL impairment is present across different tasks, the vulnerability of learning in different modalities (acoustic, visual) and domains (verbal, non-verbal) is still not clear in DLD. There is evidence that this impairment is present both in the acoustic (Hsu et al., 2014; Lukács and Kemény, 2014; Lammertink et al., 2020a) and in the visuo-motor (Lum et al., 2014; Mayor-Dubois et al., 2014) modalities. Evans et al. (2009) have demonstrated with acoustically presented linguistic and non-linguistic word segmentation tasks that the SL impairment of DLD children is equally present in both verbal and nonverbal domains.

Despite the growing body of evidence for SL deficits in individuals with DLD across multiple modalities and domains, it is still not clear how these difficulties relate to the severity and nature of the linguistic impairment. While Evans et al. (2009) found an association between statistical segmentation abilities and vocabulary size, other studies failed to observe a significant relationship between SL and language and literacy skills (expressive morphosyntax, receptive vocabulary) in children with DLD (Lammertink et al., 2020a, auditory verbal non-adjacent dependency learning task; Hedenius et al., 2011, alternating serial reaction time paradigm). While the efficiency of learning was not associated with language skills in this latter study, the consolidation of the learned structures has been shown to be related to grammatical abilities of DLD children (Hedenius et al., 2011).

It is important to highlight that most SL studies in DLD rely on sequence learning tasks, such as the serial reaction time, artificial grammar learning, non-adjacent dependency learning or statistical segmentation tasks. Results from these different studies, together with a few direct comparisons of sequential vs. nonsequential SL in DLD (Hsu and Bishop, 2014; Lukács and Kemény, 2014) suggest that sequential learning is more vulnerable in DLD, and the sequential SL impairment is present across different tasks.

To summarize these findings, there is considerable evidence for SL impairment in DLD, mostly based on online sequence learning on the serial reaction time task and on offline SL measures in artificial grammar learning or statistical segmentation tasks. Since offline measures mostly rely on grammatical well-formedness judgments that participants have to make after the training phase, these tasks provide information regarding the result of the learning process, and such measures are potentially biased by memory and metacognitive abilities. Recently, there has been increasing focus on online SL tasks, which—in contrast with the offline tasks—measure learning not after, but during the training phase, and have the potential to offer insight into the process of learning (for more details on online SL measures, see Siegelman et al., 2018). Online measures seem to provide more sensitive and reliable indices of learning than offline methods (Batterink et al., 2015; Lammertink et al., 2019; Lukics and Lukács, 2021), however currently there are only a few studies of online linguistic SL. In a recent study, Lammertink et al. (2020a) have shown with RT measurements in an online target detection task that children with DLD are less effective in learning acoustic non-adjacent dependencies than typically developing (TD) peers. At that same time, in another study (Lammertink et al., 2020b) the authors found no evidence for impaired SL in DLD in an online visual segmentation task. Our study was motivated by these controversial findings, together with methodological concerns for relying on offline measures.

### The Current Study

The current study was designed to investigate statistical sequence learning in children with DLD across modalities and domains with online and offline measures with an adapted version of the statistical segmentation paradigm (Saffran et al., 1996a).

The aims of our study were: (1) to test SL capacity in children with and without DLD; (2) to compare the sensitivity of different online (accuracy and reaction time) and offline (two-alternative forced choice and production) measures; and (3) to compare the efficiency of SL in the visual and acoustic modalities in DLD.

1. Based on previous research (e.g., Evans et al., 2009; Hsu et al., 2014; Lukács and Kemény, 2014; Lammertink et al., 2020b), we hypothesized that individuals with DLD would show impaired learning and lower performance in the SL tasks than the control group, in both the visual and acoustic modalities.
2. In most SL experiments, learning is investigated by relying on an offline two-alternative forced choice (2AFC) task (e.g., Evans et al., 2009; Hsu et al., 2014). 2AFC tasks can be used in multiple modalities and domains, but they rely on declarative memory and metalinguistic awareness, which may bias results, especially in children with atypical development. This raises the possibility that no learning is observed in DLD (e.g., Evans et al., 2009; Hsu et al., 2014) on such measures because other factors mask learning. For this reason, we also included measures of online accuracy and RT changes, and we expected them to be more direct and more sensitive indices of learning, showing significant learning in both DLD and TD. Together with online measures, we constructed our offline tests to be more sensitive, too: we used trials of varying difficulty in the 2AFC tasks (three types of comparisons are applied with both bigram and trigram sequences), which makes the task more sensitive to individual differences and we also included a production measure. We predicted that indices from all three measures (online, offline 2AFC, production) would show less effective learning in DLD than in TD. Since we expected online measures to be more sensitive to learning indices than offline measures, we expected to see significant learning in both modalities in both groups with the online indices, even where the offline tasks are not sensitive enough to show a learning effect.
3. Previous empirical findings indicate that individuals with DLD are characterized with SL impairments in both the visual (Lum et al., 2014; Mayor-Dubois et al., 2014) and in the acoustic (Hsu et al., 2014; Lukács and Kemény, 2014; Lammertink et al., 2020a) modalities. Although the SL deficit in DLD seems to be present across modalities, it is also important to note that DLD is primarily an impairment of spoken language comprehension and production, and many studies have found difficulties with auditory, but not so much with visual processing in DLD (see Leonard, 2014). Although Obeid et al. (2016) in their meta-analysis found no moderating effect of modality on the SL impairment in DLD, they also stress that studies rely on diverse methods and different tasks do not necessarily measure the same underlying construct. There are still only a few studies (e.g., Gabriel et al., 2015) that have compared SL across multiple modalities in DLD with the same paradigm. We planned to extend research in this area by testing participants with the same segmentation task in both the visual and acoustic modalities to see whether the SL impairment is equally severe in both modalities, or it is differentially affected by modality-specific constraints of SL (Frost et al., 2015) and the vulnerability of acoustic, but not of visual processing in DLD. We expected to see impaired SL in DLD in both modalities, and we hypothesized that the severity of this impairment will differ across modalities, and we will observe a more pronounced deficit in the acoustic modality.

## Materials and Methods

### Participants

Thirty-six native Hungarian-speaking children participated in the study. Children with DLD (*n* = 18) were recruited *via* a special school for children with language and speech difficulties. Since the Hungarian diagnostic system relies on the ICD-10, in which DLD is not a unique category, we recruited children that had a previous diagnosis of Expressive language disorder (F80.1), Mixed receptive-expressive language disorder (F80.2) or Developmental disorder of speech and language, unspecified (F80.9) by the Expert and Rehabilitation Committee on Cognitive Capacities of the local Pedagogical Professional Services. Since there are no standardized diagnostic or screening tools for DLD in Hungarian for this age group, we were not able to further confirm the presence of DLD in children with language tests, and based the assignment of children to the DLD group solely on the above official diagnoses. Children from two other schools without a diagnosis of DLD or any other developmental disorder were included in the control group of TD children. Inclusion criteria were: (a) nonverbal IQ score (measured by Raven’s Colored Progressive Matrices) above 85; (b) normal or corrected to normal vision; (c) normal hearing; and (d) no history of behavioral or psychiatric disorders. Typically developing control participants (*n* = 18) were recruited from two primary schools in Budapest. Groups were matched individually on age and sex (for demographic data see Table 1).

**Table 1:** Demographic data for the developmental language disorder (DLD) and typically developing (TD) groups.

	DLD (*n* = 18)	TD (*n* = 18)
Sex	m: 13; f: 5	m: 13; f: 5
Age mean (SD)	12.41 (1.77)	12.32 (1.46)
IQ mean (SD)	104.06 (12.13)	n.a.

*Note: Participants were matched on sex and age (*t*_(32)_ = 0.178, *p* = 0.860), IQ score are only available for the DLD group*.

### Stimuli

**Figure 1:**
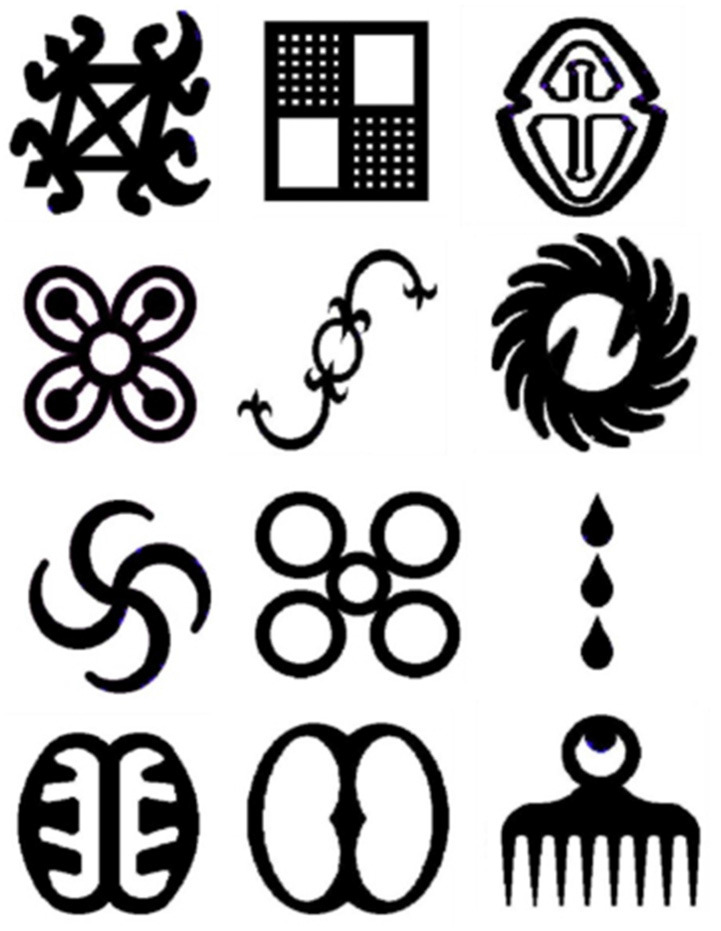
Stimuli of the visual non-verbal segmentation task ordered into triplets.

The segmentation tasks were adapted from Saffran et al.’s (1996a; 1996b; 1999) seminal studies. In the original paradigm, participants were presented with a continuous stream of 12 CV syllables organized into four trisyllabic words so that transitional probabilities (TPs) across syllables were 1 within words and 0.33 across word boundaries. After listening to the training stream, participants (8 month old babies and adults as well) were able to distinguish between syllable triplets forming words vs. syllable triplets spanning word boundaries cued only by TPs between the syllables. In the current experiment, in the acoustic segmentation task, the syllables were adopted to the phonotactic rules of Hungarian (cé /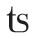
e:/, de /dε/, gá /ga:/, go/go/, ha /h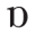/, ki /ki/, mü /my/, pe /pε/, sá ∫a:/, tu /tu/ta/t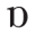/, vi /vi/), and combined into four 3-syllable nonsense words (*vösápo, hucéde, mésoki, takögá*). To avoid coarticulation effect, syllables were recorded in isolation and manipulated in Praat (Boersma and Weenink, 2021) to have a pitch of 125 Hz. The duration of syllables was 400 ms and they were presented with an ISI of 50 ms, which resulted in a presentation rate of 450 ms. In the visual task, similarly to previous studies (e.g., Bertels et al., 2012; Parks et al., 2020), 12 non-figurative monochrome symbols were applied and combined into four visual triplets (see Figure 1). The duration of symbol presentation was 800 ms with 200 ms long ISIs, resulting in a presentation rate of 1,000 ms.

### Segmentation Tasks

**Figure 2:**
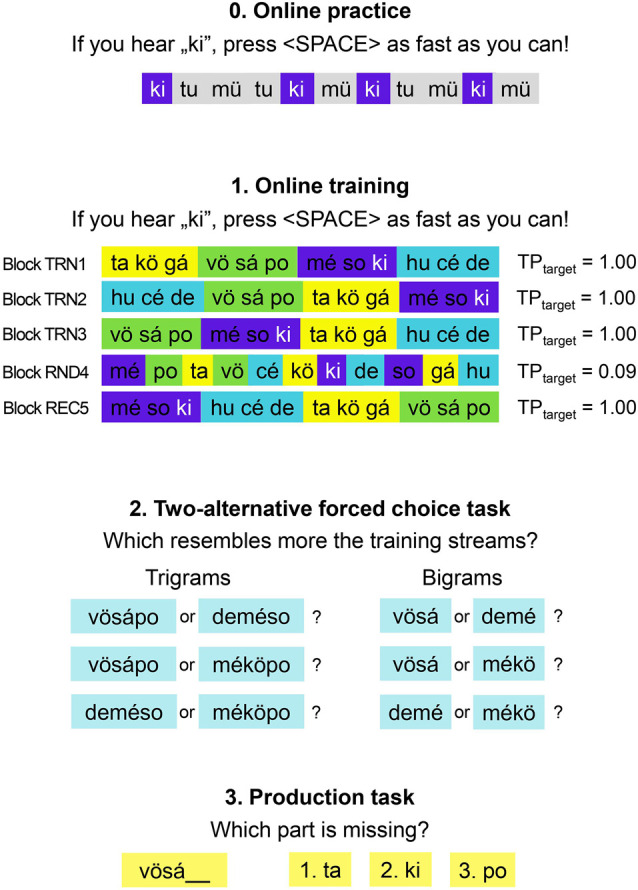
Task procedure.

Both tasks consisted of a training phase followed by a test phase with two different tasks (see Figure 2). During the training phase, participants were exposed to five unsegmented streams in five blocks. Four of the streams (Blocks 1–3 and Block 5) contained the units (syllables/symbols) in structured order: items were organized into triplets, and the four triplets occurred in a random order with the only constraint that the same sequence could not be immediately repeated. Block 4 was a random stream, in which items followed each other randomly, with the constraint that the same item could not occur twice subsequently. The streams did not provide any prosodic or other cues to segmentation, and word (triplet) boundaries were indicated only by the TPs between the items. In the structured streams, TPs were 1 between items that occurred next to each other within one of the four triplets, TPs were 0.33 at the boundaries of triplets, since the last item of a structured sequence could be followed by any of the three initial items of the three other triplets, and TPs were 0 between those units that never occurred next to each other.

The task began with a short familiarization phase, in which children were familiarized with the task and with the stimuli. At the beginning, three items were alternating in a 28 s long stream in random order with the same timing parameters as in the experiment (see section “Stimuli”), and children were instructed to respond to each item by pressing button “A”. In the next phase, children had to respond to a given target by pressing the spacebar and to foils by pressing button “A”. This practice stream contained nine targets and the criterion for proceeding (and the child understanding the task) was a spacebar press for at least six but not more than 13 items (regardless of whether these button presses were made to targets or not). After completing this phase, children were allowed to go ahead to the experimental task. If a child performed outside this range, they had two more attempts at this practice phase before they were excluded from the experiment. Two children with DLD were excluded from the sample because of failure to achieve criterion in the practice phase, yielding a sample size of 18 participants. At the next step, the task stimuli were presented individually: the visual symbols visually and the syllables both visually and acoustically.

During the training phase, participants performed a target detection task. The target was the last item of one of the four triplets counterbalanced between participants. Children were instructed to respond to the target by pressing the spacebar, while in every other case they had to push the button “A” on the keyboard through the five training blocks. The blocks consisted of 180 target items: each item occurred 15 times in both the structured and random streams. During this phase of the task, accuracy and RT measures were collected.

The online training phase was followed by a 2AFC task, in which bigram and trigram pairs were presented and children were instructed to choose the one that is more familiar to what they previously heard (the stream of the training phase). To make the task more sensitive to individual differences, the 36 in the 2AFC test trials varied in difficulty and in length. The comparison types are presented in Table 2.

**Table 2:** Comparison types in the two-alternative forced choice (2AFC) task.

Length	Target	Target TP	Distractor	Distractor TP	*N* of Trials
Bigrams	word	1	partword	0.33	6
	word	1	random	0	6
	partword	0.33	random	0	6
Trigrams	word	1, 1	partword	0.33, 1	6
	word	1, 1	random	0, 0	6
	partword	0.33, 1	random	0, 0	6

After completing the 2AFC task, participants performed a production task, in which incomplete sequences were presented and their task was to complete them with one of three items. The 12 trials varied in the location and/or identity of the missing item within the sequence. Each triplet occurred three times in the task.

### Procedure

Participants were tested with the informed consent of their parents together with their own informed consent, in accordance with the principles set out in the Declaration of Helsinki. The study was approved by the United Ethical Review Committee for Research in Psychology (EPKEB-2018/87). Tasks were administered in small groups of 2–4 in silent classrooms in the schools; if three or four children were tested, two experimenters supervised the testing. Both segmentation tasks were coded in PsychoPy 3, and data were recorded *via* the Pavlovia online platform. Children completed the two segmentation tasks in two sessions (one task in each session), with at least a week apart; the order of the tasks was counterbalanced.

The study started with 20 children with DLD, and—as mentioned above—two of them were excluded because they could not complete the practice phase of the tasks. Two participants of the DLD group left the school before the second session could have been completed, for this reason one of them missed the acoustic and the other one missed the visual task. They and their control pairs were only included in the analysis of the segmentation tasks they completed. Two other DLD participants failed to give any response in one or more of the training blocks in the acoustic task, therefore together with their control pairs they were excluded from the analyses of the online acoustic measures. In the control group, one participant could not finish with the visual task because of technical problems, so he and his DLD pair were excluded from the analysis of the visual segmentation task. Together with these changes, 16 children with DLD and 16 controls were included in the analyses of both online and offline results of the visual task, while the analyses of the acoustic tasks were conducted with the inclusion of 15–15 pairs of children in the online and 17–17 pairs in the offline tasks.

## Results

### Segmentation Tasks

In the online target detection tasks, we measured the accuracy of responses and RTs of accurate button presses for targets. Responses within a 1,800 ms time window from −600 ms to 1,200 ms from stimulus onset time were considered in the analyses. We filtered outliers by participant: RTs outside the 1.5 interquartile range beyond the first and third quartiles of their own RTs were excluded from the analyses.

#### Acoustic Verbal Segmentation

**Figure 3:**
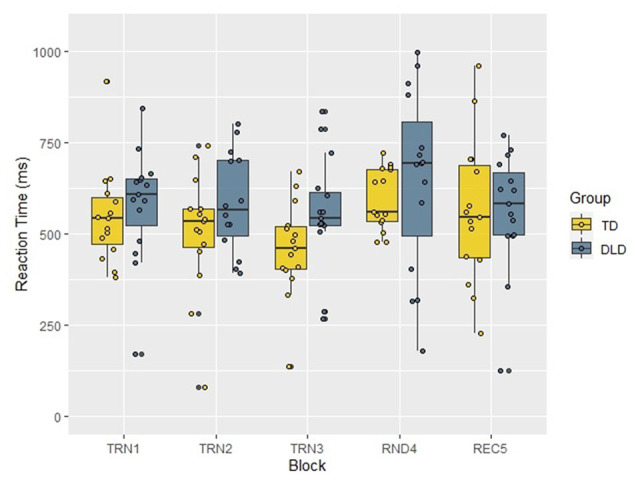
Median RTs by Block and Group in the acoustic verbal target detection task. TD, typically developing children; DLD, children with Developmental language disorder; TRN1, First training block; TRN2, Second training block; TRN3, Third training block; RND4, Random block with disrupted structure; REC5, Recovery block.

To investigate the progress of learning through blocks, we calculated median RTs and accuracy rates for each block (see Figure 3). We conducted a repeated measures ANOVA on the median RTs with Block as within-subject and Group as between-subject factors. According to the Mauchly’s test, the assumption of sphericity has been violated ($\chi_{\left(9\right)}^{2}$ = 25.59, *p* = 0.002), therefore Greenhouse-Geisser correction was applied (*ɛ* = 0.704). The results showed a significant main effect of Block (*F*_(2.82,78.82)_ = 3.01, *p* = 0.038, *η*^2^ = 0.097). Tests of within-subjects contrast revealed that median RTs in Block 3 and Block 4 differed significantly, with large effect sizes from median RTs of the previous blocks (Block 2 > Block 3: *F*_(1,28)_ = 6.11, *p* = 0.020, $\eta_{p}^{2}$ = 0.179; Block 3 < Block 4: *F*_(1,28)_ = 9.86, *p* = 0.004, $\eta_{p}^{2}$ = 0.260). At the same time, neither Block 1 vs. Block 2 (*F*_(1,28)_ = 0.015, *p* = 0.902, $\eta_{p}^{2}$ = 0.001), nor Block 4 vs. Block 5 (*F*_(1,28)_ = 2.04, *p* = 0.165, $\eta_{p}^{2}$ = 0.068) comparisons showed a significant difference. The main effect of Group was not significant (*F*_(1,28)_ = 1.80, *p* = 0.190, $\eta_{p}^{2}$ = 0.060) and neither was the interaction between the two factors (*F*_(2.82,78.82)_ = 1.06, *p* = 0.370, $\eta_{p}^{2}$ = 0.036). Since these null results were associated with small or moderate effect sizes, they might be caused by small statistical power, and this way, effect sizes do not help with our conclusions concerning the absence of a significant difference in these comparisons.

**Figure 4:**
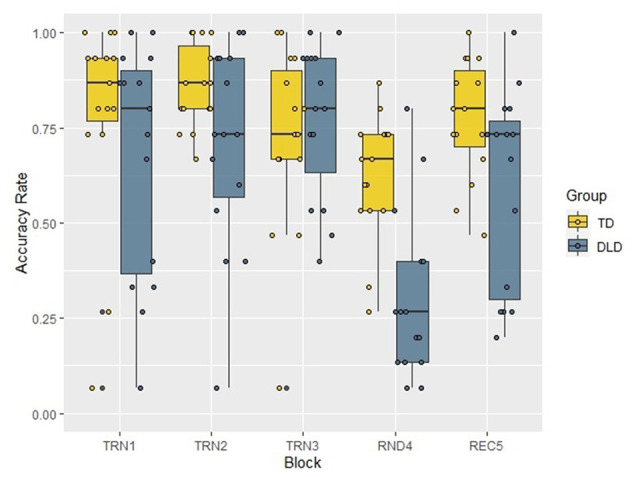
Accuracy rates by Block and Group in the acoustic verbal target detection task. TD, typically developing children; DLD, children with Developmental language disorder; TRN1, First training block; TRN2, Second training block; TRN3, Third training block; RND4, Random block with disrupted structure; REC5, Recovery block.

We investigated the pattern of accuracy rates in the two groups (distributions presented in Figure 4) in a similar repeated measures ANOVA with Block as a within-subject factor and Group as a between-subject factor. The Mauchly’s test revealed that the assumption of sphericity was satisfied ($\chi_{\left(9\right)}^{2}$ = 10.23, *p* = 0.333), and there was no need for correction. The analysis revealed the significant main effect of Block (*F*_(4,112)_ = 17.55, *p* < 0.001, *η*^2^ = 0.385) and the within-subject contrast analyses showed that the random block differed significantly, with large effect sizes from the previous and the following ones (Block 3 > Block 4: *F*_(1,28)_ = 37.29, *p* < 0.001, $\eta_{p}^{2}$ = 0.571; Block 4 < Block 5: *F*_(1,28)_ = 27.44, *p* < 0.001, $\eta_{p}^{2}$ = 0.495), while Block 1 vs. Block 2 (*F*_(1,28)_ = 1.61, *p* = 0.215, $\eta_{p}^{2}$ = 0.054) and Block 2 vs. Block 3 (*F*_(1,28)_ = 0.80, *p* = 0.380, $\eta_{p}^{2}$ = 0.028) comparisons did not show significant differences. The main effect of Group (TD > DLD: *F*_(1,28)_ = 5.13, *p* = 0.031, *η*^2^ = 0.155), and the interaction of Block and Group was also significant (*F*_(4,112)_ = 4.91, *p* = 0.001, *η*^2^ = 0.149) and these comparisons reached large effect sizes as well. The follow-up analyses, applying Bonferroni correction and establishing significance level at 0.01, revealed that the accuracy rates of the two groups differed significantly in Block 4 (*t*_(28)_ = −4.52, *p* < 0.001, *d* = 1. 65), while in Block 1 (*t*_(28)_ = −1.09, *p* = 0.284, *d* = 0.40), Block 2 (*t*_(18.60)_ = −2.09, *p* = 0.05, *d* = 0.76), Block 3 (*t*_(28)_ = 0.65, *p* = 0.522, *d* = 0.24) and Block 5 (*t*_(22.94)_ = −2.26, *p* = 0.034, *d* = 0.83) there was no difference between the groups. To investigate the interaction between Block and Group further, we also tested the change of accuracy rates with two repeated measures ANOVAs in the two groups separately. Both tests showed significant effect of Block (TD: *F*_(2.61,36.55)_ = 3.92, *p* = 0.020, *η*^2^ = 0.219, DLD: *F*_(4,56)_ = 19.65, *p* < 0.001, *η*^2^ = 0.584). In the follow up analyses, significance levels were adjusted to 0.01. Accuracy in Blocks 1 vs. Block 2, and in Block 2 vs. Block 3 did not differ in either group. However, Blocks 3 and 4 only differed in the DLD group (with lower accuracies in Block 4). Performance in Block 5 compared to Block 4 was higher in both groups. Although the comparison of these blocks showed only a tendency level difference in the TD group, the analysis reached a large effect size, which indicates that learning was evident in both groups. Results for within-subject contrast analyses are reported in Table 3.

**Table 3:** Within-subjects contrast analyses by groups, for the online accuracy measures in the acoustic verbal segmentation task.

		*df*	*F*	*p*	*η* ^2^
TD	Block 1 vs. 2	1	1.27	0.279	0.083
	Block 2 vs. 3	1	4.85	0.045	0.257
	Block 3 vs. 4	1	1.55	0.234	0.099
	Block 4 vs. 5	1	6.98	0.019^+	0.333
DLD	Block 1 vs. 2	1	0.39	0.544	0.027
	Block 2 vs. 3	1	3.03	0.104	0.178
	Block 3 vs. 4	1	89.70	<0.001**	0.865
	Block 4 vs. 5	1	21.92	<0.001**	0.610

Significance level was adjusted to 0.01 by using the Bonferroni correction. ^+^*p* < 0.1; **p <0.01.

To investigate learning effects in offline 2AFC measures, we conducted one-sample *t*-tests separately in the two groups on the offline indices to establish the presence of learning. In the 2AFC task, each trial contained two items (one target and one distractor), therefore chance level was at 0.5; in the Production task, participants had to choose between three items (one target and two distractors), and consequently, the test value of chance level analysis was at 0.33. Descriptive statistics of offline measures and results of *t*-tests are reported in Table 4. While TD children showed a significant learning effect in both tasks, the DLD group performed at chance in the 2AFC task and showed only a tendency level learning effect in the production task.

**Table 4:** Descriptives and comparative statistics of groups performance relative to chance level in the offline acoustic verbal tasks.

	DLD (*n* = 17)			TD (*n* = 17)		
	*mean (SD)*	*t*	*p*	*mean (SD)*	*t*	*p*
2AFC	0.48 (0.10)	−0.87	0.396	0.54 (0.07)	2.20	0.043*
Production	0.38 (0.11)	1.90	0.076^+^	0.50 (20)	3.32	0.004**

^+^*p* < 0.1; *p <0.05; **p <0.01.

**Figure 5:**
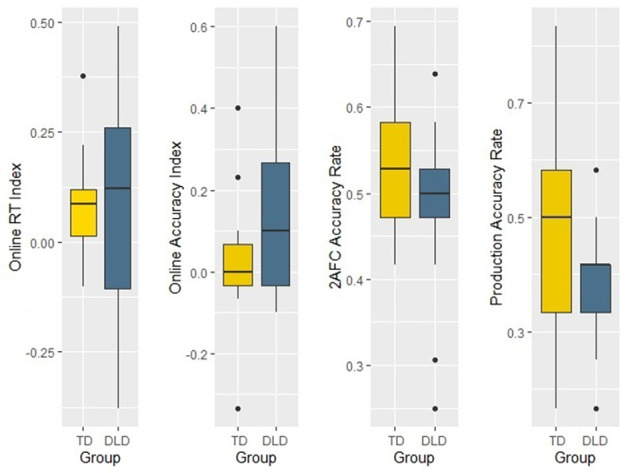
Learning indices of the two groups in the acoustic verbal segmentation task.

To compare learning efficiency in the two groups, we defined four learning indices: (1) The RT index was calculated as the mean of the deviations of median RT in the random block compared to the adjacent ones (RT index = ((Block 4 − Block 3) + (Block 4 − Block 5))/2). (2) Similarly, an accuracy index was calculated as the mean of the deviations of accuracy rate in Block 4 (random condition) relative to accuracy rates in Block 3 and Block 5 (Accuracy index = ((Block 3 − Block 4) + (Block 5 − Block 4))/3). (3) The offline 2AFC index was defined by the accuracy rate on the forced choice task and (4) the accuracy rate in the production task yielded the offline production index. To examine whether the DLD group differed from the TD group in learning efficiency, we performed four independent-samples *t*-tests on the above indices (see Table 5 and Figure 5). The Accuracy index differed significantly between the two groups with higher values in the DLD group, while the 2AFC and the Production tasks showed the advantage of the control group, with marginally significant group differences and moderate effect sizes. The RT index did not show any significant difference.

**Table 5:** Independent-samples *t*-test analyses for each learning index of the acoustic verbal segmentation task.

	DLD	TD				
	*mean (SD)*	*mean (SD)*	*df*	*t*	*p*	*d*
RT index	87.27 (263.19)	84.87 (113.86)	28	0.03	0.974	0.14
Accuracy index	0.38 (0.19)	0.12 (0.24)	28	3.22	0.003**	0.18
2AFC	0.48 (0.09)	0.54 (0.07)	32	−2.05	0.049*	0.70
Production	0.38 (0.11)	0.50 (0.20)	32	−2.04	0.052	0.70

*p <0.05; **p <0.01.

#### Visual Non-verbal Segmentation

**Figure 6:**
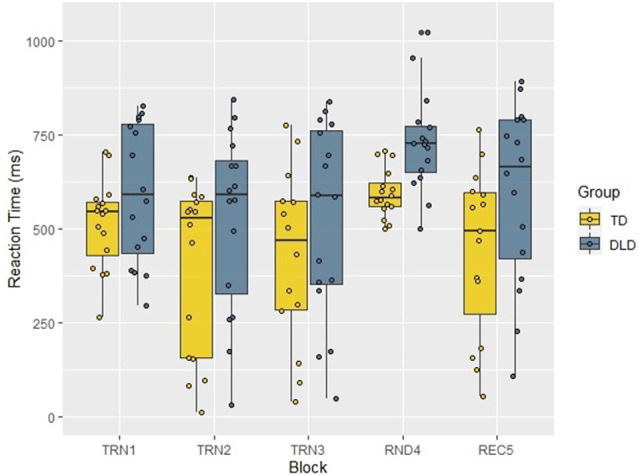
Median RTs by Block and Group in the visual non-verbal target detection task. TD, typically developing children; DLD, children with Developmental language disorder; TRN1, First training block; TRN2, Second training block; TRN3, Third training block; RND4, Random block with disrupted structure; REC5, Recovery block.

Similarly to the analysis of the acoustic verbal task, we conducted a repeated measures ANOVA on the median RTs of the visual non-verbal segmentation task too (see Figure 6), with Block as a within-subject and Group as a between-subject factor. Since the assumption of sphericity was violated ($\chi_{\left(9\right)}^{2}$ = 44.51, *p* < 0.001), we applied the Greenhouse-Geiser correction (*ɛ* = 0.571). The analysis revealed significant main effects with large effect sizes for Block (*F*_(2.28,68.48)_ = 17.58, *p* < 0.001, *η*^2^ = 0.369) and Group (DLD > TD *F*_(1,30)_ = 4.57, *p* = 0.041, *η*^2^ = 0.132), while the interaction of these factors (*F*_(2.28,68.48)_ = 0.86, *p* = 0.442, *η*^2^ = 0.028) was not significant. Follow-up tests of within-subjects contrasts on adjacent blocks showed that RTs differed from each other significantly in three pairs of adjacent Blocks (Block 1 > Block 2: *F*_(1,30)_ = 13.34, *p* = 0.001, $\eta_{p}^{2}$ = 0.308; Block 3 < Block 4: *F*_(1,30)_ = 29.75, *p* < 0.001, $\eta_{p}^{2}$ = 0.498; Block 4 > Block 5: *F*_(1,30)_ = 20.26, *p* < 0.001, $\eta_{p}^{2}$ = 0.403), and all of these comparisons reached large effect sizes. The comparison of Block 2 and Block 3 did not show any significance (*F*_(1,30)_ = 0.54, *p* = 0.469, $\eta_{p}^{2}$ = 0.018), and the low effect size may indicate a power issue.

**Figure 7:**
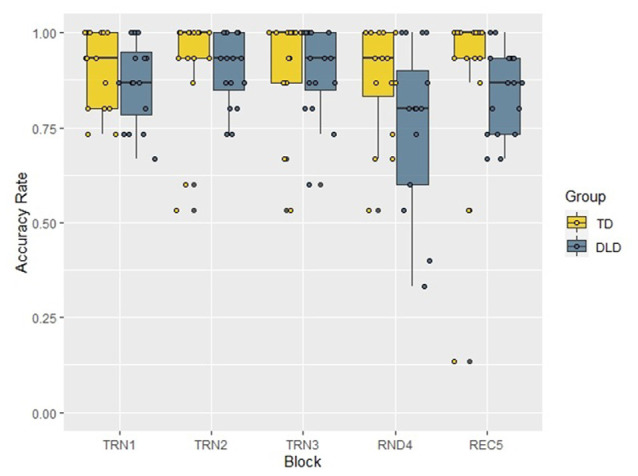
Accuracy rates by Block and Group in the visual non-verbal target detection task. TD, typically developing children; DLD, children with Developmental language disorder; TRN1, First training block; TRN2, Second training block; TRN3, Third training block; RND4, Random block with disrupted structure; REC5, Recovery block.

The same analysis was performed with accuracy rate as the dependent variable (see Figure 7). The Mauchly test showed that the assumption of sphericity was not satisfied ($\chi_{\left(9\right)}^{2}$ = 28.76, *p* = 0.001), therefore we applied the Greenhouse-Geisser correction (*ɛ* = 0.719). The ANOVA revealed that the Block had a significant main effect on accuracy (*F*_(2.88,86.29)_ = 3.58, *p* = 0.018, *η*^2^ = 0.107), while either the main effect of Group (*F*_(1,30)_ = 1.85, *p* = 0.184, *η*^2^ = 0.058), or the interaction of Block and Group (*F*_(2.88,86.29)_ = 1.01, *p* = 0.392, *η*^2^ = 0.032) did not reach significance. The within-subject contrast analyses showed a significant accuracy rate difference between the random block and the previous one with a large effect size (Block 3 > Block 4: *F*_(1,30)_ = 8.83, *p* = 0.006, *η*^2^ = 0.227), but in the other blocks, accuracy rates did not differ significantly from those in the previous blocks, and effect sizes were small or moderate in all of these comparisons (Block 1 vs. Block 2: *F*_(1,30)_ = 1.11, *p* = 0.301, *η*^2^ = 0.036; Block 2 vs. Block 3: *F*_(1,30)_ = 0.03, *p* = 0.873, *η*^2^ = 0.001; Block 4 vs. Block 5: *F*_(1,30)_ = 1.98, *p* = 0.169, *η*^2^ = 0.062).

Descriptive statistics for the offline measures together with results of the one-sample *t*-tests are reported in Table 6. Both groups showed significant learning in the offline production task, but while the TD children performed above chance level in the 2AFC task as well, the group children with DLD performed at chance in this task.

**Table 6:** Descriptives and comparative statistics of groups performance relative to chance level in the offline visual non-verbal tasks.

	DLD (*n* = 16)			TD (*n* = 16)		
	*mean (SD)*	*t*	*p*	*mean (SD)*	*t*	*p*
2AFC	0.54 (0.10)	1.51	0.153	0.64 (0.14)	4.04	0.001***
Production	0.50 (0.21)	3.13	0.007**	0.58 (0.23)	4.39	0.001***

**p <0.01; ***p <0.001.

**Figure 8:**
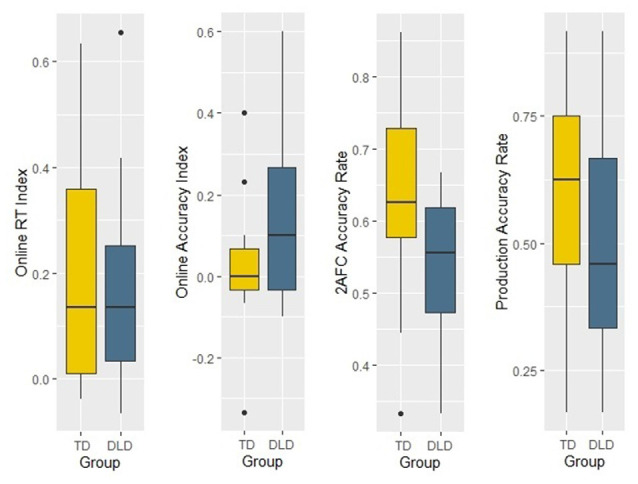
Learning indices of the two groups in the visual non-verbal segmentation task.

To investigate whether there are any differences between groups in learning efficiency, we examined the same learning indices as in the acoustic verbal task version (see above for the descriptions of the indices). We conducted four independent-samples *t*-tests on the four learning indices. Descriptive statistics of performance in the different groups and the results of group comparisons are reported in Table 7 (see Figure 8). Results indicate that TD participants performed significantly better in the 2AFC task than the DLD group with a large effect size, however the RT, Accuracy and Production indices did not show any significant difference between the groups.

**Table 7:** Independent-samples *t*-test analyses for each learning index of the visual non-verbal segmentation task.

	DLD	TD				
	*mean (SD)*	*mean (SD)*	*df*	*t*	*p*	*d*
RT index	170.38 (187.18)	208.81 (225.67)	30	−0.524	0.604	0.19
Accuracy index	0.11 (0.21)	0.03 (0.15)	30	1.323	0.196	0.47
2AFC	0.54 (0.10)	0.64 (0.14)	30	−2.376	0.024*	0.84
Production	0.50 (0.21)	0.59 (0.23)	30	−1.123	0.271	0.40

*p <0.05.

#### Learning Across Modalities and Domains

To test whether the SL impairment in DLD differs across modalities and domains, we conducted two repeated measures ANOVAs on the learning indices of the two offline tasks. In the first ANOVA we included the 2AFC index as dependent variable, Modality as within-subject variable and Group as between-subjects variable. The ANOVA revealed significant main effect of Modality (visual > acoustic: *F*_(1,28)_ = 13.422, *p* = 0.001, *η*^2^ = 0.324) and Group (TD > DLD: *F*_(1,28)_ = 6.956, *p* = 0.013, *η*^2^ = 0.042), but there was no significant interaction between Modality and Group (*F*_(1,28)_ = 1.223, *p* = 0.278, *η*^2^ = 0.324). In the second analysis we included the Production task index as dependent variable, Modality as within-subject and Group as between-subjects variable. The analysis revealed a significant main effect of Modality (visual > acoustic: *F*_(1,28)_ = 4.771, *p* = 0.037, *η*^2^ = 0.146) and a tendency level effect of Group (TD > DLD: *F*_(1,28)_ = 3.062, *p* = 0.091, *η*^2^ = 0.099), while the interaction of these variables was not significant (*F*_(1,28)_ = 0.034, *p* = 0.854, *η*^2^ = 0.001). Since there were no group differences in online learning in either modality, we did not compare online measures across the acoustic and visual modalities.

## Discussion

The present study was designed to investigate SL abilities in children with and without DLD across two modalities (acoustic verbal and visual non-verbal) with a statistical segmentation task. Based on previous studies (Evans et al., 2009; Hsu et al., 2014; Lukács and Kemény, 2014; Haebig et al., 2017; Lammertink et al., 2020a), we expected to see an SL impairment in DLD in both modalities. We relied on three tasks in measuring SL: (1) an online target detection task, which is not biased by decision making and metalinguistic abilities; (2) an offline 2AFC task with a high number of trials varying in difficulty to increase the sensitivity of the task; and (3) an offline production task. Beyond investigating whether children with DLD show SL impairments across multiple modalities and domains, we also aimed to compare the sensitivity of different online and offline learning indices.

In the online phases of both the acoustic and visual tasks, we observed a learning effect in both groups, and found SL to be as efficient in DLD as in typically developing children. This was reflected in decreased accuracy and increased RTs in the random block (Block 4) relative to the neighboring structured blocks (Blocks 3 and 5). In the acoustic verbal task, the learning effect was only present between Blocks 3 and 4 (in RTs), and was nominally present but not statistically significant between Blocks 4 and 5, probably due to low statistical power and/or the tiring of participants.

The acoustic online accuracy index was bigger in the DLD group, but this seemingly surprising result was due to a significant drop in accuracy rates in DLD in the random block, together with accuracy rates remaining relatively high in TD even with unstructured stimuli. Therefore in this case a higher accuracy index does not reflect more efficient learning in DLD. The nonverbal visual task showed a similar pattern: while children in the DLD group had slower RTs in general, no SL deficit was reflected in RT patterns, as indicated by non-significant differences in RT patterns between DLD participants and controls, and online learning was present in both groups. Similarly, both groups showed evidence of learning and a similar learning effect in accuracy rates.

By having a task that makes online tracking of learning possible, more fine-grained, reliable and valid measures of learning became available, allowing tracking the speed as well as the level of learning. Measuring continuous changes in RT throughout the task allows us to examine how learning proceeds in different groups (i.e., young vs. older children and adults and in neurodevelopmental disorders), and compare the time course of learning developmentally and in different populations, testing differences in speed of attainment, and whether learning happens gradually or suddenly. This could reveal differences potentially hidden in post experimental offline indices: two groups may show similar learning on a task, but learning could be faster in one than the other. We exploited this possibility, and beyond investigating the efficiency of SL by contrasting performance in the unstructured and structured blocks, we also monitored the process of learning through the first three training blocks. Our results revealed that both groups showed increasing efficiency in predicting targets through the training blocks (Block 1-Block 3), which suggests that SL proceeded continuously in both groups. Only the acoustic verbal accuracy measures revealed a difference in the progress of SL between the groups: while the TD group showed near ceiling performance across all blocks, which led to the masking of learning in the TD group, the DLD group showed a learning effect in the comparison of Block 3 vs. Block 4 and Block 4 vs. Block 5.

While we found efficient learning and similar learning efficiency in the DLD and TD groups on online measures, offline tasks showed a different pattern and impaired learning in DLD on several measures. In the offline acoustic verbal tasks, there was no evidence of learning for children with DLD while the TD group showed significant learning and higher performance on both 2AFC and production measures. In the visual nonverbal task, the DLD group showed evidence of learning on the production task, but their performance was at chance in the 2AFC task. The TD group showed significantly higher performance and a significant learning effect on the 2AFC task, and they also showed evidence of learning on the visual production index. Taken together, these findings only partially support our first hypothesis: efficient learning and no SL deficit was observed in DLD on online measures, but we found impaired learning relative to controls on offline measures in both the acoustic verbal and visual nonverbal tasks.

This pattern and the dissociation in performance between online and offline measures supports our second hypothesis, i.e., that online measures would be able to better detect learning than offline measures. Online measures based on both accuracy and RT showed that children with DLD are able to extract patterns that are cued only by transitional probabilities in both acoustic and visual modalities. At the same time, we observed no learning on the offline 2AFC task in DLD. A possible explanation for this dissociation is that the metacognitive and short-term memory components of the forced choice tasks are especially challenging for children with DLD, and may mask learning in these offline tasks, while they do not bias SL performance in the target detection tasks.

This pattern is in line with Lammertink et al.’s (2020b) findings relying on an online target detection task, showing similar statistical segmentation of visually presented triplets in groups of children with and without DLD. At the same time, target detection showed group differences in non-adjacent dependency learning in another study from the same lab (Lammertink et al., 2020a) where they found impaired SL in DLD by relying on RT measures from an acoustic non-adjacent dependency learning task. As they point out, non-adjacent dependency learning is cognitively more demanding than detecting adjacent dependencies, and evidence of impaired SL in online tasks can be a function of task difficulty too. Further studies are needed to explore which online and offline measures are suited for testing variation in SL within and across populations. The sensitivity of both online and offline tasks should be increased by the use of items that differ in complexity. In the current study, we found that the use of trials differing in length and difficulty increased the sensitivity of the 2AFC task. Incorporating this type of item variation in the online task, which in its current form included a single bisyllabic transition as a target, could lead to a more difficult online task and more sensitive online measures as well.

In our third hypothesis, we predicted that the SL impairment in DLD will be more pronounced in the acoustic verbal than the visual nonverbal task. The patterns of results were similar across modalities: both groups showed significant learning in the online measures, and the SL impairment in DLD was evidenced by at least one of the offline tasks. Direct comparison of group performances across modalities did not reveal any difference between the severity of the SL impairment in DLD. Although this finding is in line with observations of Obeid et al. (2016), who reported no effect of modality on the severity of SL impairment in DLD in their meta-analysis, the small effect size of the analysis of the Production task indicates that this null result could be a result of low statistical power, and the pattern of results—which showed a SL impairment in DLD in the acoustic modality and no group difference in the visual modality—indicate potentially greater vulnerability of acoustic SL in DLD. Also, the analysis of the 2AFC task showed no difference in the severity of the SL impairment in DLD across modalities, and the large effect size of the test indicates no power issue that could bias this result. In the acoustic modality, both the 2AFC and the production task showed the advantage of the control group relative to TD children with moderate effect sizes, while in the visual modality, only the 2AFC task proved to be more difficult for children with DLD than for controls. Although the analysis of the visual 2AFC task reached a large effect size, it does not necessarily reflect a SL impairment, since we cannot exclude the potential influence of metacognitive skills or short-term memory capacity, especially because in the Production task we did not detect a SL impairment in DLD in this modality. Since our two tasks seemed to have differed in difficulty (see offline results for TD children) and in the domain of stimuli as well, these results only suggest a more severe impairment in the acoustic modality, and call for further studies with parallel tasks in different modalities and domains. Our results suggest that the SL deficit in DLD is present across multiple modalities, which results in successful, but less effective SL in DLD than in TD.

Examining SL across modalities and domains in DLD may provide new insights into questions of domain generality of principles and modality specific constraints of SL and also extend our knowledge about the cognitive profile of the disorder. The specificity of language impairments in DLD has long been questioned, and impairments in domain general cognitive functions (among them SL) have been amply documented, but the question of domain generality or specificity of SL are still open. We found a SL impairment in DLD in both the acoustic and visual modalities (with moderate effect sizes in the acoustic and with large effect size in the visual modality), which confirms previous studies showing SL impairment in DLD across modalities and domains (Evans et al., 2009; Lukács and Kemény, 2014; Obeid et al., 2016). As discussed in the previous paragraph, since the SL impairment of children with DLD was detected by two measures in the acoustic, and only by one (which might be biased by metacognitive and short-term memory functions) in visual modality, our results also suggest a more severe deficit in the acoustic modality. This pattern is expected based on the presence of problems with auditory processing in DLD, and the suggestion that SL operates with modality and domain specific constraints (Frost et al., 2015).

Our study has potential limitations. First, the number of participants was relatively low (targeting children with atypical development in a small country during the pandemic), relying on a large sample would have increased statistical power. Second, we investigated SL by measuring the recognition of transitional probabilities in adjacent dependencies. As mentioned above, a paradigm that involves more complex patterns (such as non-adjacent dependency learning or artificial grammar learning tasks with more complex sequences and/or involving abstraction and generalization) may provide deeper insight into the SL difficulties of children with DLD through accuracy and RT changes. Including more diverse trials (i.e., words, partwords and non-words) in the online task would increase the efficiency of the online measures further. Another potential limitation of the study is that while we were aware of the metacognitive component of the offline tasks, we did not control for the contribution of such metacognitive factors to performance in the offline task. While offline measures indicated the presence of an SL impairment in DLD across modalities, online learning indices only showed impaired learning in the acoustic verbal task. In contrast, neither RT, nor accuracy measures revealed a SL deficit in DLD in the online visual non-verbal task. Based on this pattern of results in the offline vs. online tasks, we cannot exclude the possibility that differences in the visual 2AFC task were mediated by metacognitive or short-term memory components of the task. By controlling for these components, we could get a better picture of the nature of the SL impairment in DLD. Further studies addressing these limitations are needed to investigate the SL in children with and without DLD.

## Conclusion

Our results show that when learning is tracked online, children with DLD are as efficient as typically developing children in extracting acoustic verbal and visual nonverbal patterns in a segmentation task based solely on transitional probabilities. However, we observed an SL impairment in DLD in both modalities and domains on post-training measures that involve metacognitive functions. These results confirm previous findings of an SL impairment in DLD, and also provide insight into the process of learning by the use of online measures. Online accuracy and RT measures yield sensitive SL indices, and show evidence of learning where factors outside SL potentially mask learning in offline tasks. By relying on three different tasks, our results highlight that different SL measures diverge in their sensitivity to learning and to detecting SL differences across populations. They imply that the combined use of different measures and trials with variable complexity provides a better picture of learning efficiency and a deeper understanding of SL in different populations.

## Data Availability Statement

The raw data supporting this article is available at the following link: https://osf.io/h3vu2/?view_only=56e8b5eb17fe4c80864ff0b02790602b.

## Ethics Statement

The studies involving human participants were reviewed and approved by United Ethical Review Committee for Research in Psychology (EPKEB-2018/87). Written informed consent to participate in this study was provided by the participants’ legal guardian/next of kin.

## Author Contributions

ÁL, KL and DD contributed equally to the design of the study, data collection, statistical analyses, and publication. All authors contributed to the article and approved the submitted version.

## Conflict of Interest

The authors declare that the research was conducted in the absence of any commercial or financial relationships that could be construed as a potential conflict of interest.

## Publisher’s Note

All claims expressed in this article are solely those of the authors and do not necessarily represent those of their affiliated organizations, or those of the publisher, the editors and the reviewers. Any product that may be evaluated in this article, or claim that may be made by its manufacturer, is not guaranteed or endorsed by the publisher.
